
JOURNAL INFORMATION
==============================
NLM Title Abbreviation: ChemistryOpen
Journal ID: ChemistryOpen
NLM Journal ID: 101594811
Journal ID: open

ChemistryOpen

EISSN: 2191-1363
Publisher: Wiley

ARTICLE INFORMATION
==============================
PMCID: PMC3703811
PMID: 24551543
DOI: 10.1002/open.201390014
Article version: 1
Subjects: News

Spotlights on our sister journals: ChemistryOpen 3/2013

Print publication date: 2013 Jun
Electronic publication date: 2013 Jun 20
Volume: 2
Issue: 3
First page: 84
Last page: 87
Copyright: Copyright © 2013 WILEY-VCH Verlag GmbH & Co. KGaA, Weinheim
Copyright year: 2013
License: Re-use of this article is permitted in accordance with the Creative Commons Deed, Attribution 2.5, which does not permit commercial exploitation.
License URL: https://creativecommons.org/licenses/by/2.5/

==============================
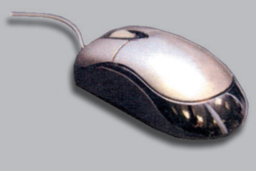

On these pages, we feature a selection of the excellent work that has recently been published in our sister journals. If you are reading these pages on a computer, click on any of the items to read the full article. Otherwise please see the DOIs for easy online access through Wiley Online Library.

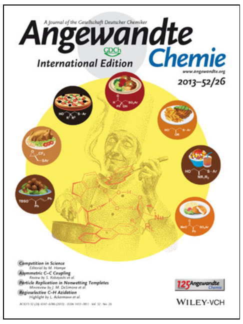

### Nanoparticle Assembly

*I. Kosif, M. Cui, T. P. Russell, T. Emrick**

**Triggered In situ Disruption and Inversion of Nanoparticle-Stabilized Droplets**

**Emulsion inversion**, from water-in-oil (w/o) to oil-in-water (o/w), was accomplished by employing tetrahydropyran-containing ligands that undergo facile deprotection, converting the nanoparticles from hydrophobic to hydrophilic. These ligand-tailored nanoparticles were used to prepare w/o emulsions that were disrupted, and inverted, to o/w systems simply by lowering the solution pH. The inversion process could be triggered by light using a photoacid generator.

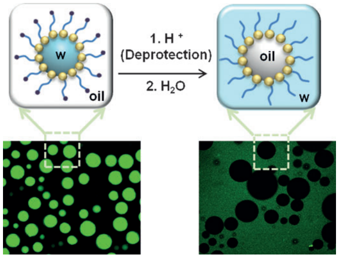

*Angew. Chem. Int. Ed.*
DOI: **10.1002/anie.201302112**

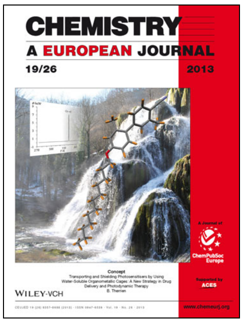

### Computer Chemistry

*S.-F. Chen, N. Ferré, Y.-J. Liu**

**QM/MM Study on the Light Emitters of Aequorin Chemiluminescence, Bioluminescence, and Fluorescence: A General Understanding of the Bioluminescence of Several Marine Organisms**

**See jellyfish in a new light**: The origin and differences in the chemiluminescence, bioluminescence, and fluorescence of the proteins of the aequorin jellyfish were theoretically investigated by performing hybrid quantum mechanics and molecular mechanics methods combined with a molecular dynamics method. The findings are important for understanding the bioluminescence of jellyfish and other marine organisms (see figure).

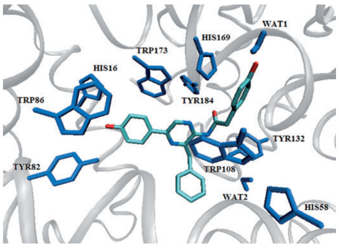

*Chem. Eur. J.*
DOI: **10.1002/chem.201300678**

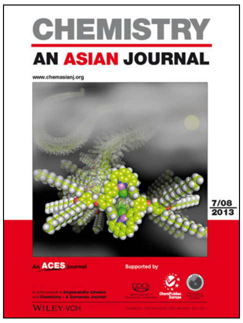

### Hybrid Complexes

*K. Naoda, H. Mori, A. Osuka**

**Gold$^{III}$–Iridium$^{III}$ Hybrid Complexes of Hexaphyrin(1.1.1.1.1.1)**

**Au revoIR**: Hexaphyrin Au$^{III}$–Ir$^{III}$ complexes were prepared from the reaction of a mono-Au$^{III}$ [26]hexaphyrin complex with $[IrCl(cod)]_2$ followed by treatment with pyridine. *N*-Fusion reaction of this complex gave a syn-doubly *N*-fused hexaphyrin as the major product, which displayed antiaromatic properties such as strong paratropic ring current, a small HOMO–LUMO gap and a low-energy forbidden absorption. The oxidation of this fused product with $MnO_2$ afforded an oxidatively cleaved product.

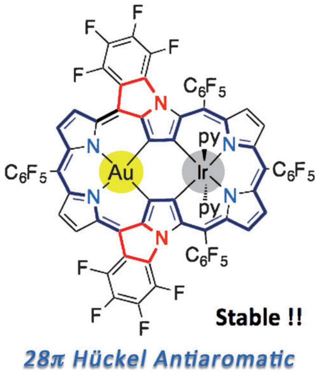

*Chem. Asian J.*
DOI: **10.1002/asia.201300320**

© 2013 Wiley-VCH Verlag GmbH & Co. KGaA, Weinheim

ChemPubSoc Europe

### Directed Evolution

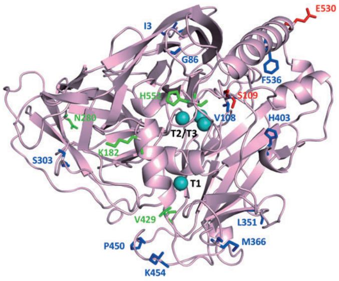

*P. Torres-Salas, D. M. Mate, I. Ghazi, F. J. Plou, A. O. Ballesteros, M. Alcalde**

**Widening the pH Activity Profile of a Fungal Laccase by Directed Evolution**

**Unnatural selection**: A fungal laccase was tailored by directed evolution to be active at neutral/alkaline pH. After five generations, the final mutant showed a broader pH profile while retaining 50 to 80% of its activity at neutral pH.

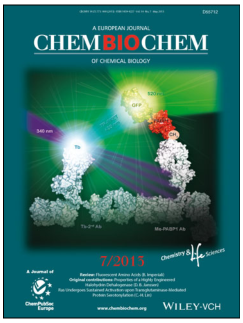

*ChemBioChem*
DOI: **10.1002/cbic.201300102**

### Carbon Electrodes

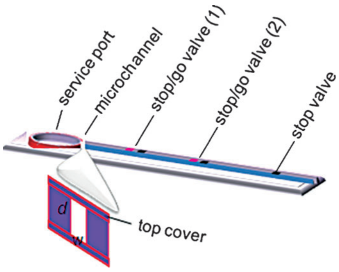

*A. P. Washe, P. Lozano, D. Bejarano, I. Katakis**

**Eletrochemically Actuated Stop–Go Valves for Capillary Force-Operated Diagnostic Microsystems**

**Nanoporous graphitic carbon** electrodes display a unique electrowetting behavior that depends on specific ion effects. When incorporated into a microfluidic channel, these superhydrophobic electrodes facilitate sequential stop–go fluidic operations, which are directly applicable to diagnostic microsystems.

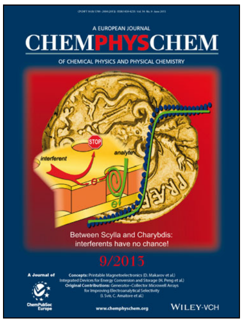

*ChemPhysChem*
DOI: **10.1002/cphc.201300042**

### Subtype-Selective Ligands

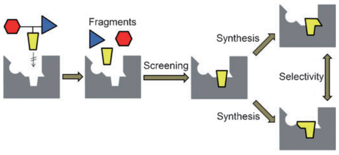

*A. J. Thompson, M. H. P. Verheij, J. E. van Muijlwijk-Koezen, S. C. R. Lummis,* R. Leurs, I. J. P. de Esch*

**Structure–Activity Relationships of Quinoxaline-Based 5-HT$_3$A and 5-HT$_3$AB Receptor-Selective Ligands**

**One atom makes the difference**: By using a fragment screen as an efficient route to design novel ligands, we identified quinoxalines as high-affinity binders to 5-HT$_3$ receptors. New synthesis routes allowed the development a series of compounds able to distinguish 5-HT$_3$A receptors from 5-HT$_3$AB receptors, revealing that a single atom is sufficient to change the selectivity profile of a compound.

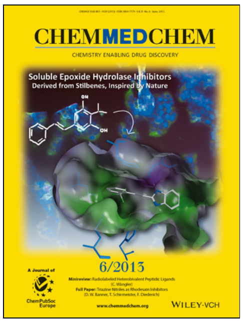

*ChemMedChem*
DOI: **10.1002/cmdc.201300032**

### Batteries

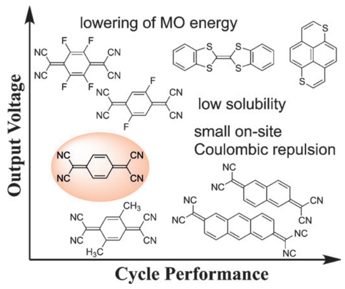

*S. Nishida, Y. Yamamoto, T. Takui,* Y. Morita**

**Organic Rechargeable Batteries with Tailored Voltage and Cycle Performance**

**Made to order**: Rechargeable batteries are fabricated by using organic electron acceptors and donors as active cathode materials. Their output voltage and cycle performance can be tuned by organic chemistry techniques. The output voltages are linked to both the redox potentials and the energy levels of the frontier molecular orbitals of the cathode materials, enabling to predict the output voltage at an early stage of the design.

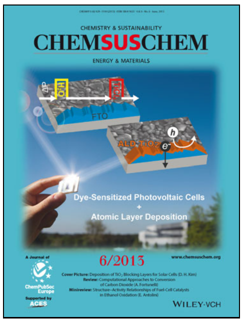

*ChemSusChem*
DOI: **10.1002/cssc.201300010**

© 2013 Wiley-VCH Verlag GmbH & Co. KGaA, Weinheim

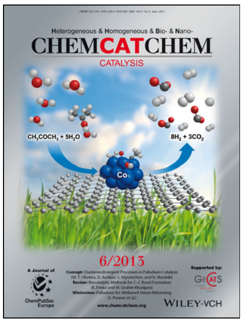

### Oxidative Dehydrogenation

*W. Gan, D. J. M. Snelders, P. J. Dyson, G. Laurenczy**

**Ruthenium(II)-Catalyzed Hydrogen Generation from Formic Acid using Cationic, Ammoniomethyl-Substituted Triarylphosphine Ligands**

**Proceed with cation:** New Ru$^{II}$ catalysts for aqueous-phase HCOOH decomposition into $H_2$ and $CO_2$ that use a series of oligocationic, ammoniomethyl-substituted triarylphosphine ligands are developed. Excellent activities are achieved, that is, TOFs of 1950 h$^{-1}$ and TONs of over 10 000.

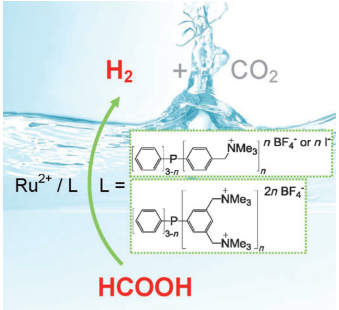

*ChemCatChem*
DOI: **10.1002/cctc.201200782**

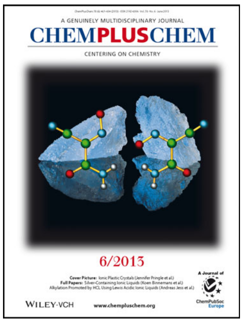

### Tracking Multifunctional Nanoparticles

*S. N. Goonewardena,* H. Zong, P. R. Leroueil, J. R. Baker, Jr.*

**Bioorthogonal Chemical Handle for Tracking Multifunctional Nanoparticles**

**Getting a handle on it**: An innovative strategy for tracking multifunctional nanoparticles in biological systems is presented that simplifies the syntheses and can be used to track the nanoparticles in vitro and in vivo. The strategy is based on in situ click chemistry ligation of fluorescent reporters to the nanoparticle scaffold through copper-catalyzed azide–alkyne cycloaddition (see figure).

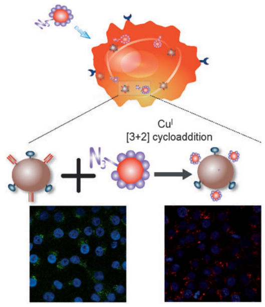

*ChemPlusChem*
DOI: **10.1002/cplu.201300007**

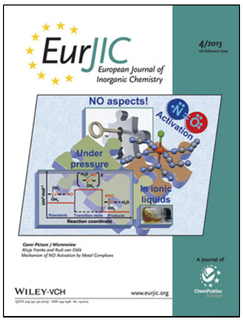

### Cobalamins

*D. T. Walker, R. S. Dassanayake, K. A. Garcia, R. Mukherjee, N. E. Brasch**

**Mechanistic Studies on the Reaction of Nitrocobalamin with Glutathione: Kinetic Evidence for Formation of an Aquacobalamin Intermediate**

Kinetic studies on the reaction of nitrocobalamin ($NO_2$Cbl) with glutathione show that glutathionylcobalamin (GSCbl) is formed via an aquacobalamin ($H_2$OCbl$^+$) intermediate. This reaction pathway is demonstrated by independently determining individual rate constants for each step.

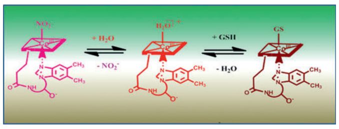

*Eur. J. Inorg. Chem.*
DOI: **10.1002/ejic.201300254**

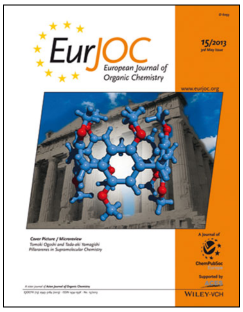

### Macrocyclic β-Sheets

*R. Spencer, K. H. Chen, G. Manuel, J. S. Nowick**

**Recipe for β-Sheets: Foldamers Containing Amyloidogenic Peptide Sequences**

Recipe for β-Sheets: Combine one *N*-methyl-amino acid, two δ-linked ornithine turn units, and nine α-amino acids by solid-phase peptide synthesis. Cyclize in solution, deprotect, and purify by RP-HPLC. Confirm folding by $^1$H NMR ROESY and magnetic anisotropy studies. Serve to laboratories interested in studying and inhibiting amyloid aggregation or studying β-sheet assembly.

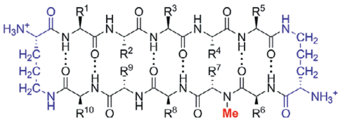

*Eur. J. Org. Chem.*
DOI: **10.1002/ejoc.201300221**

© 2013 Wiley-VCH Verlag GmbH & Co. KGaA, Weinheim

### Cobyrinic Acid Derivatives

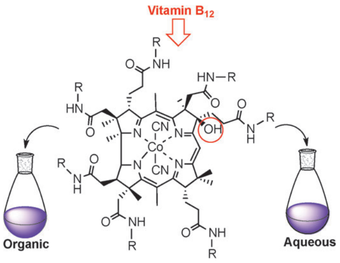

*Asian J. Org. Chem.*
DOI: **10.1002/ajoc.201300051**

*K. ó. Proinsias, M. Giedyk, Ł. Banach, D. Rutkowska-Zbik, D. Gryko**

**Selectively Modified Cobyrinic Acid Derivatives**

**Vitamin $B_{12}$ - more possibilities**: An efficient method for the aminolysis of cobyrinic acid derivatives catalyzed by a benzotriazole/DBU system is described. The synthesized cobinamide derivatives can be easily functionalized at either the *c* or *meso* position. $(CN)_2Cby(III)OMe_7$ is prone to hydroxylation at the C8 position. The location of hydroxylation was confirmed through NMR analysis and the reactivity of this position was analyzed by computational methods.

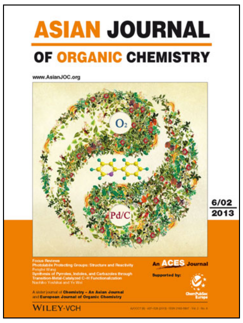

### Quinine

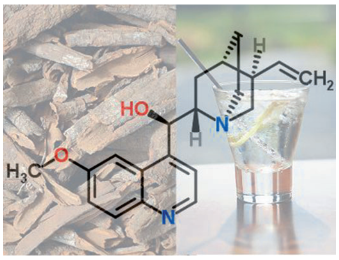

*ChemViews magazine*
DOI: **10.1002/chemv.201300056**

*K. Roth and S. Streller*

**From Pharmacy to the Pub - A Bark Conquers the World: Part 1**

The bark of the Cinchona tree is probably the most valuable drug the Americas gave the world. It yielded the first effective remedy for malaria, one of the most dangerous of infectious diseases. Its main component, quinine, is still used as a medication - but also for tonic water and Bitter Lemon. K. Roth and S. Streller, Berlin, look at the history and, of course, chemistry of this important substance.

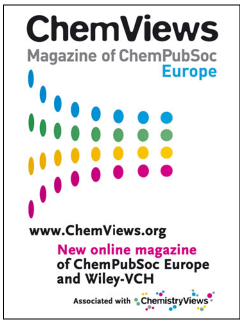

© 2013 Wiley-VCH Verlag GmbH & Co. KGaA, Weinheim
